# Assessment of Deep Convolutional Neural Network Models for the Classification of Benign Fibro‐Osseous Lesions of the Jaws

**DOI:** 10.1002/cre2.70244

**Published:** 2025-10-16

**Authors:** Paniti Achararit, Chawan Manaspon, Chavin Jongwannasiri, Kraisorn Sappayatosok, Thanaphum Osathanon, Ekarat Phattarataratip

**Affiliations:** ^1^ Princess Srisavangavadhana College of Medicine, Chulabhorn Royal Academy Bangkok Thailand; ^2^ Biomedical Engineering Institute Chiang Mai University Chiang Mai Thailand; ^3^ College of Dental Medicine Rangsit University Pathum Thani Thailand; ^4^ Center of Excellence for Dental Stem Cell Biology, Department of Anatomy, Faculty of Dentistry Chulalongkorn University Bangkok Thailand; ^5^ Department of Oral Pathology, Faculty of Dentistry Chulalongkorn University Bangkok Thailand

**Keywords:** cemento‐osseous dysplasia, cemento‐ossifying fibroma, convolutional neural network, deep learning, diagnosis, fibro‐osseous lesions, fibrous dysplasia

## Abstract

**Objectives:**

Benign fibro‐osseous lesions (BFOL) constitute a group of pathologic entities with marked overlapping histopathologic features but are diverse in nature and clinical behaviors. Accurate diagnoses of BFOLs necessitate clinical‐pathological correlations, which are paramount for their appropriate management. Recent research indicates the potential utility of artificial intelligence in diagnostic pathology. Here, we aimed to assess the performance of the deep convolutional neural network (DCNN) models for BFOL classification and investigate its impact on the diagnostic performance of oral pathologists.

**Material and Methods:**

Microscopic slides from 68 patients diagnosed with cemento‐ossifying fibroma (COF), fibrous dysplasia (FD), and cemento‐osseous dysplasia (COD) were collected. The image patches from each slide were processed, augmented, and used to train and validate the five pre‐trained DCNN models for BFOL classification. The best‐performing model was selected to evaluate its diagnostic performance on the testing data set, compared with experienced oral pathologists.

**Results:**

The InceptionV3 model showed the highest and most balanced overall performance in BFOL classification. It demonstrated the highest accuracy (96.7%) in classifying COF, followed by COD (83.3%), and FD (80.0%), respectively. The model accuracy in identifying COF was greater than the average performance of pathologists (90.0%). However, pathologists performed better in classifying COD (87.2%) and FD (95.0%). With DCNN assistance, pathologists significantly improved the accuracy, sensitivity, and specificity in distinguishing BFOLs while reducing the average diagnosis time.

**Conclusions:**

The DCNN model has the potential to be developed as an auxiliary tool, assisting pathologists in diagnosing BFOLs. Through ongoing refinements, artificial intelligence assistance can aid pathologists in enhancing the accuracy and efficiency of BFOL diagnosis.

## 1 Introduction

Benign fibro‐osseous lesions of the jaws (BFOL) represent a group of pathologic conditions affecting gnathic bones. They share similar microscopic features, consisting of a variable proportion of mineralized materials intermixed with fibrous connective tissue. These lesions span from the disorders of bone dysplasias to benign neoplasms and demonstrate vastly different clinical behaviors and prognoses (Waldron 1993). The most common BFOLs in respective order of prevalence are cemento‐osseous dysplasia (COD), fibrous dysplasia (FD), and cemento‐ossifying fibroma (COF) (El‐Mofty 2014; Slootweg 1996).

COD is a non‐neoplastic fibro‐osseous lesion occurring exclusively within the tooth‐bearing areas of the alveolar bones. It is mostly asymptomatic, non‐expansile, and no treatment is required (Olgac et al. 2021). FD is a developmental disturbance of bone growth associated with *GNAS1* mutation. It can arise from any site but shows a propensity for the craniofacial bones. Clinically, FD is usually diagnosed during childhood or adolescence due to the noticeable bone expansion. However, following the patient's skeletal maturation, the lesion becomes stable. Therefore, its treatment mainly involves surgical recontouring of the lesion to correct the cosmetic deformity (Burke et al. 2017). In contrast, COF is a benign odontogenic neoplasm with a broad age range. If left untreated, COF can cause significant expansion, and complete enucleation is required (Titinchi and Morkel 2016).

Due to each condition's distinct clinical behaviors and treatment options, accurately diagnosing BFOLs is crucial for optimal treatment planning and patient outcomes. Pathologic examination is considered the gold standard for diagnosis. Nevertheless, these lesions exhibit significantly overlapping microscopic features, and distinguishing BFOLs has been an area of difficulty for pathologists, particularly on the small incisional specimens (Phattarataratip et al. 2014). Clinical, radiographic, and pathologic correlations are essential for the accurate diagnosis of these conditions.

COD and COF share a number of similar characteristics histopathologically. Both lesions are composed of diverse types of mineralized tissues, including woven bone, lamellar bone, and cementum‐like materials, at varying proportions (Su et al. 1997). FD generally consists predominantly of curvilinear‐shaped woven bone trabeculae. However, those involving the jaw bones are known to develop lamellar differentiation as they mature. In addition, the surrounding fibrous connective tissue of all BFOLs may be variably cellular, depending on the types and maturation stages of the lesions (Slootweg 1996). As a result, pathologists relied on subtle microscopic clues present in each specimen, and the clinical and radiographic correlation is essential for rendering definitive diagnoses (Soluk‐Tekkesin et al. 2022).

Recent advances in computational pathology have enabled the development of a laboratory digital workflow (Verghese et al. 2023). With this technology, artificial intelligence (AI) has emerged as a potentially helpful tool in assisting pathologists in various tasks (Herdiantoputri et al. 2024; Lopez‐Janeiro et al. 2022; Pirayesh et al. 2024). Researchers have developed deep learning (DL) models to help classify/grade tumors, detect structures of interest, such as metastatic deposits, mitotic figures, or predict treatment response, associated mutations, and others (Katayama et al. 2024; Komura and Ishikawa 2019). In the field of oral pathology, multiple roles of DL have been intensively examined. Yang et al. (2022) demonstrated that DL could diagnose oral squamous cell carcinoma with 0.98 sensitivity and 0.92 specificity and helped pathologists reach the diagnosis faster with increased accuracy than when working alone. In odontogenic lesions, AI models were tested and showed potential for diagnosing as well as predicting the prognosis of odontogenic keratocyst (Cai et al. 2024) and distinguishing between ameloblastoma and ameloblastic carcinoma (Giraldo‐Roldan et al. 2023). Furthermore, Peng et al. (2024) established the DL model that can detect and grade oral epithelial dysplasias with 81.3% accuracy, which could help increase their grading reproducibility among pathologists. Despite several concerns regarding the generalization or the lack of a rationale‐based explanation of its prediction, AI emerges as a promising adjunctive apparatus in diagnostic pathology (Alajaji et al. 2024; Araújo et al. 2023).

In the present study, we aimed to investigate the performance of the deep convolutional neural network (DCNN) models for the classification of three common BFOLs based on the histopathologic findings and to assess its potential contribution to the diagnostic performance of oral pathologists in distinguishing these lesions in clinical practice.

## 2 Methods

### 2.1 Data Set Preparation

**Figure 1:**
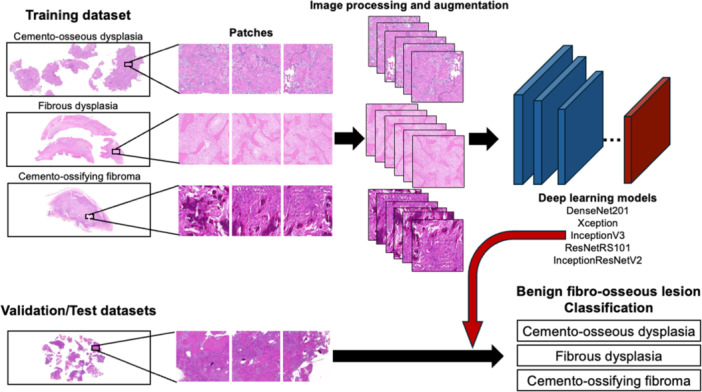
Overview of the data acquisition and DCNN classification framework.

This study was approved by the Faculty of Dentistry, Chulalongkorn University Human Research Ethics Committee (DCU 2022‐089) and performed according to the Declaration of Helsinki. The overview of the data acquisition scheme and model architecture is depicted in Figure 1. The data set comprised microscopic slides collected from 68 BFOL patients diagnosed with COD (30 cases), FD (19 cases), and COF (19 cases) from the Department of Oral Pathology at the Faculty of Dentistry, Chulalongkorn University, between 2012 and 2021. All microscopic slides were reviewed together with patients’ clinical and radiographic records. Cases with pronounced tissue artifacts or significant secondary inflammation/infection were excluded. The diagnoses, based on the 5th edition of the WHO Classification of Head and Neck Tumours, were verified by board‐certified oral and maxillofacial pathologists, who were not included in the pathologist and DCNN assistance experiment and used as ground truth.

A total of 1571 histopathologic images were obtained from H&E‐stained slides at 100× magnification using an Olympus CX31 microscope paired with a Canon EOS 600D EOS Digital SLR camera. The photomicrographs representing each pathologic lesion were captured at 100× magnification in high resolution to ensure detailed visibility of the histopathologic features. Images were further categorized into three datasets: 494 for training (24 cases), 296 for validation (14 cases), and 781 for testing (30 cases).

### 2.2 Image Pre‐Processing and Augmentation

Pre‐processing of the histopathologic images was performed to enhance the quality and uniformity of the data set. Initially, the images were resized to a uniform dimension of 512 × 512 pixels. Subsequently, the pre‐processing incorporated several steps of image augmentation techniques to optimally prepare each image for training. These augmentation procedures, including rescaling, horizontal/vertical flips, rotation/width shift/height shift/channel shift ranges, and fill mode, were carried out using the “ImageDataGenerator” class from the Keras library (Perez and Wang 2017).

Custom random erasing augmentation was also employed to enhance model robustness by simulating potential occlusions and variability inherent in clinical settings (Zhong et al. 2020). Each augmented image underwent a random erasing operation, where the area for erasing was randomly determined, accounting for 2%–20% of the total image area with an aspect ratio between 0.2 and 2. A rectangle, defined by the previously calculated dimensions, was placed within the image randomly. This area was filled with random pixel values, creating an “erased” zone that introduces artificial occlusions.

The validation data set underwent a less rigorous pre‐processing regimen, primarily involving the rescaling of pixel values. This approach ensured that the transformations applied during model validation mimicked those during the training phase and provided an unbiased assessment of the model's performance on standardized data.

### 2.3 Deep Convolutional Neural Network Training

Five distinct DCNN architectures were selected to identify the optimal model for BFOL classification. Each model represented a different approach to DL‐based image analysis. The Inception family, comprising InceptionV3 (Szegedy et al. 2016), Xception (Chollet 2017), and InceptionResNetV2 (Szegedy et al. 2017), was chosen to systematically evaluate multi‐scale feature extraction strategies. InceptionV3, with its inception modules, captures features at multiple scales simultaneously. Xception replaces standard convolutions with depthwise separable convolutions, improving computational efficiency while maintaining feature extraction. InceptionResNetV2 combines the multi‐scale approach with residual connections, enhancing gradient flow and feature representation. ResNetRS‐101 (Bello et al. 2021) assesses the benefits of extremely deep networks (101 layers) enabled by residual skip connections, avoiding vanishing gradients. DenseNet201 (Huang et al. 2017) implements a different connectivity paradigm where each layer receives feature maps from all preceding layers, promoting maximum information flow and feature reuse. This diverse selection enables comprehensive evaluation of feature extraction and information processing strategies for distinguishing complex histopathologic patterns in BFOLs.

Each model was initialized with weights pre‐trained on the ImageNet data set, excluding the top layer. Specifically, a global average pooling layer was added, followed by a dense layer with three units and a SoftMax activation function. Each model was compiled with the Adam optimizer (Kingma and Ba 2015) and categorical cross‐entropy loss function. Additionally, we implemented a novel dynamic learning rate adjustment strategy, called “ResetPolicy,” alongside model checkpointing to optimize the training phases and preserve the best‐performing models.

The “ResetPolicy” adjusted the learning rate dynamically during training to alternate between specified high and low boundaries across a predetermined number of epochs (reset interval). This approach helped navigate the loss landscape, fostered quicker convergence, and avoided local minima.

### 2.4 Performance of Pathologists With DCNN Assistance

The model with the best performance metrics was selected for further analysis with pathologists to verify the applicability of the DCNN model and its impact on clinical practice. Thirty additional BFOL cases, consisting of 10 COD, FD, and COF cases, were selected as a real‐world testing data set. They were subjected to the DCNN algorithm, and the predictive results of each case were computed.

Six experienced pathologists who did not participate in the standard reference diagnoses or data set preparation were enrolled, and informed consents were obtained. They were asked to review the entire microscopic glass slides of 30 BFOL cases at a self‐controlled pace and provided one of the three BFOL diagnoses (COD, FD, and COF) for each case. In accordance with the model, the patient's clinical and radiographic history was not provided. The time each pathologist used to complete the diagnoses of the entire data set was recorded. After a 4‐week washout period, pathologists were requested to re‐examine these testing data set slides together with the predictive results from the DCNN model and provided diagnoses.

### 2.5 Statistical Analysis and the Model Performance Evaluation Metrics

The overall accuracy, precision, recall, F1‐score, receiver operating characteristic (ROC) curves, and the area under the ROC curve (AUC) were used to assess the efficacy of DCNNs in the BFOL classification. For the multi‐class classification scenario presented, each class‐specific performance metric was computed using a “one‐vs‐all” strategy, where one class was considered the positive class. In contrast, the others were grouped as negative classes. Each metric and the corresponding mathematical formula used for calculation are as follows.

For each class c,

$$\mathrm{Overall}\;\mathrm{Accuracy}=\frac{\sum_{c}\mathrm{TP}}{\mathrm{Total}\;\mathrm{number}\;\mathrm{of}\;\mathrm{prediction}}$$

$${\mathrm{Precision}}_{c}=\frac{TP_{c}}{TP_{c}+FP_{c}}$$

$${\mathrm{Recall}}_{c}=\frac{TP_{c}}{TP_{c}+FN_{c}}$$

$$F1-{\mathrm{Score}}_{c}=\frac{2\times {\mathrm{Pricision}}_{c}\times {\mathrm{Recall}}_{c}}{{\mathrm{Precision}}_{c}+{\mathrm{Recall}}_{c}}$$

where $TP_{c}$ is the number of correct predictions that an instance is a class c,

$FP_{c}$ is the number of incorrect predictions that an instance is a class c, and

$FN_{c}$ is the number of incorrect predictions where an instance of class c

is predicted as not c.

To evaluate the efficacy of DCNN as an adjunctive diagnostic tool, the AUCs, accuracy, sensitivity, specificity, and the diagnosis time that pathologists used in classifying BFOL cases with and without DCNN assistance were computed. The differences in these metrics were compared using the paired *t*‐tests. A *p* value < 0.05 was considered statistically significant. Statistical analyses were conducted using GraphPad Prism version 10.3.0.

## 3 Results

### 3.1 Performance of DCNN Models

**Figure 2:**
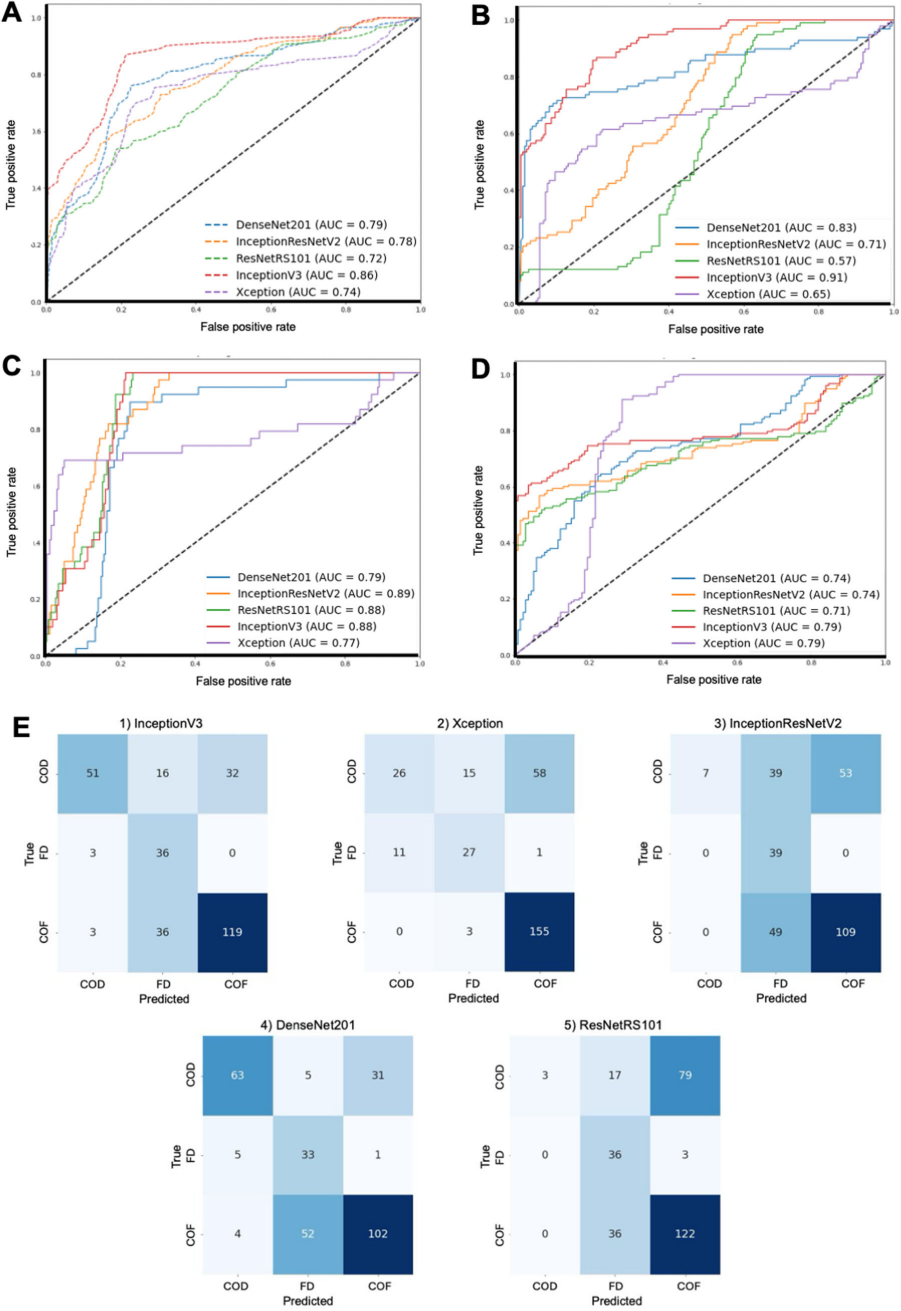
Performance of DCCN models on the validation data set in classifying BFOLs. Receiver operating characteristic curves (ROC) and the area under curves (AUC) of five DCNN models tested. (A) Average performance of all BFOL lesions, (B) COD classification, (C) FD classification, (D) COF classification, and (E) confusion matrices of five DCNN models.

The performance of five DCNN models (InceptionV3, Xception, InceptionResNetV2, DenseNet201, and ResNetRS101) in correctly classifying the histopathologic images of the validation data set into the respective categories of BFOLs was assessed using the outputs of the confusion matrices (Figure 2). Diagonal sections represented the number of images where the model prediction was similar to the actual diagnoses, whereas the off‐diagonal sections demonstrated the number of images each model misinterpreted. The darker blue highlighted the greater diagnostic accuracy of each model. In addition, the performance metrics, that is, the classification accuracy, precision, recall, and F1‐score, are summarized in Table 1.

**Table 1:** Performance metrics of each DCNN model on the BFOL validation data set.

BFOL types	Metrics (%)	Deep convolutional neural network models				
		InceptionV3	Xception	InceptionResNetV2	DenseNet201	ResNetRS101
Cemento‐osseous dysplasia	Accuracy	69.59	70.27	52.36	66.89	54.39
	Precision	89.47	70.27	100.00	87.50	100.00
	Recall	51.52	26.26	7.07	63.64	3.03
	F1‐score	65.38	38.24	13.21	73.68	5.88
Fibrous dysplasia	Accuracy	69.59	70.27	52.36	66.89	54.39
	Precision	40.91	60.00	30.71	36.67	40.45
	Recall	92.31	69.23	100.00	84.62	92.31
	F1‐score	56.69	64.29	46.99	51.16	56.25
Cemento‐ossifying fibroma	Accuracy	69.59	70.27	52.36	66.89	54.39
	Precision	78.81	72.43	67.28	76.12	59.80
	Recall	75.31	98.10	68.99	64.56	77.22
	F1‐score	77.02	83.33	68.13	69.86	67.40
All lesions	Accuracy	69.59	**70.27**	52.36	66.89	54.39
	Precision	**69.73**	67.57	66.00	66.76	66.75
	Recall	**73.05**	64.53	58.69	70.94	57.52
	F1‐score	**66.36**	61.95	42.78	64.90	43.18

*Note:* Bold values indicate the highest performance of each metric.

The Xception and InceptionV3 models exhibited the highest overall accuracy in classifying three types of BFOL at 70.27% and 69.59%, respectively, followed by the DenseNet201 (66.89%), ResNetRS101 (54.39%), and InceptionResNetV2 (52.36%). When comparing other metrics, InceptionV3 yielded the highest precision (69.73%), recall (73.05%), and F‐score (66.36%).

The ROC curves and the AUCs of these DCNN models are shown in Figure 2. The AUC values for all three types of BFOL classification were 0.86, 0.74, 0.78, 0.79, and 0.72 for the InceptionV3, Xception, InceptionResNetV2, DenseNet201, and ResNetRS101 models, respectively. The InceptionV3 model also demonstrated the highest AUC values in the COD (0.91) and COF (0.79) classifications and the second highest in classifying FD (0.88), with a slightly lower AUC value than that of InceptionResNetV2 (0.89).

**Figure 3:**
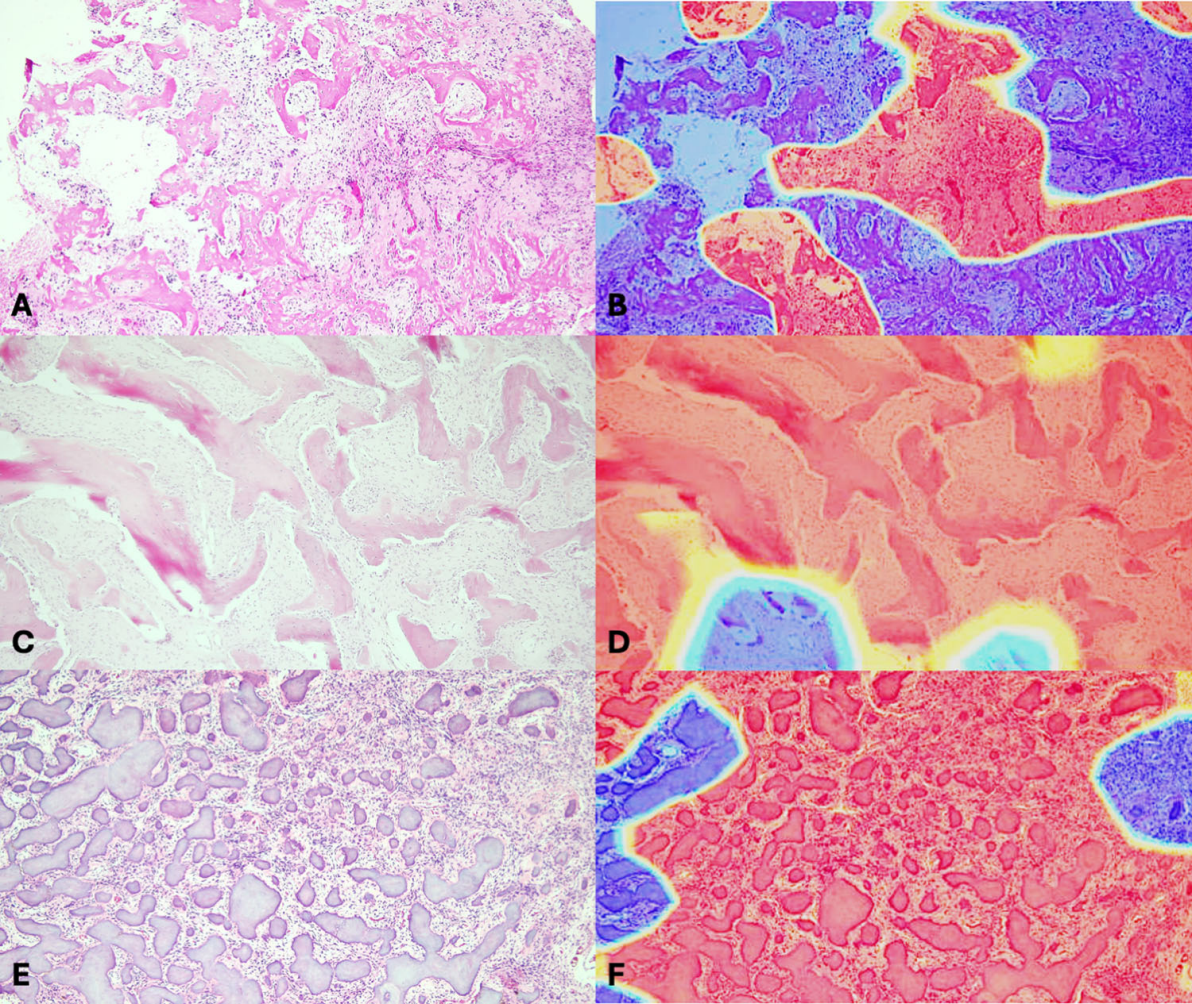
Representative gradient‐weighted class activation maps (heatmaps) showing the regions of interest of the InceptionV3 model for the classification of COD (A, B), FD (C, D), and COF (E, F).

Based on all these performance metrics, the InceptionV3 model offered the highest and most balanced performance, rendering it most suitable for clinical application. The gradient‐weighted class activation mapping (Grad‐CAM) was also generated to visualize the regions of interest that the InceptionV3 algorithm used in classifying BFOLs. Representative histopathologic images of each lesion and the corresponding heat map are presented in Figure 3. The InceptionV3 model was, therefore, selected for further analysis by human experts.

### 3.2 Diagnostic Performance of the DCNN Model in Comparison With Pathologists

To compare the performance of the DCNN model with the experienced oral pathologists, the additional test data set consisting of 30 BFOL cases was submitted to the InceptionV3 algorithm and six board‐certified pathologists for interpretation. Based on this test data set, the AUC of this DCNN model was 0.967 and demonstrated an overall 80% sensitivity and 90% specificity. When considering each lesion individually, the DCNN demonstrated the highest accuracy in classifying COF (96.7%), followed by COD (83.3%) and FD (80.0%).

When comparing the performance of DCNN and pathologists, we found that the DCNN model showed greater accuracy in identifying COF (96.7%) than the average performance of pathologists (90.0%). The sensitivity, specificity, and F1‐scores for detecting COF were 100%, 95.0%, and 95.2% for the DCNN and 83.3%, 93.3%, and 84.4% for pathologists, respectively. However, pathologists performed better in classifying COD and FD. The model's accuracy for detecting COD and FD was 83.3% and 80.0%, whereas pathologists accurately diagnosed these lesions at 87.2% and 95.0%, respectively. The DCNN demonstrated greater sensitivity but less specificity for detecting COD, while for FD lesions, the model showed inferior sensitivity but comparable specificity compared to pathologists. The detailed diagnostic performance of the DCNN model and pathologists is presented in Table 2.

**Table 2:** Diagnostic performances on the BFOL test data set of the DCNN model and pathologists working on their own.

Metrics	Cemento‐osseous dysplasia		Fibrous dysplasia		Cemento‐ossifying fibroma	
	Pathologists	Deep learning	Pathologists	Deep learning	Pathologists	Deep learning
TP	8	9	9.5	5	8.33	10
FN	2	1	0.5	5	1.67	0
TN	18.17	16	19	19	18.67	19
FP	1.83	4	1	1	1.33	1
Sensitivity (%)	80.0	90.0	95.0	50.0	83.3	100.0
Specificity (%)	90.8	80.0	95.0	95.0	93.3	95.0
PPV (%)	81.6	69.2	90.8	83.3	85.8	90.9
NPV (%)	90.8	80.0	95.0	95.0	93.3	95.0
F1‐score (%)	80.7	78.3	92.6	62.5	84.4	95.2
Accuracy (%)	87.2	83.3	95.0	80.0	90.0	96.7

Abbreviations: FN, false negative; FP, false positive; NPV, negative predictive value; PPV, positive predictive value; TN, true negative; TP, true positive.

### 3.3 Diagnostic Performance and Efficiency of Pathologists With DCNN Assistance

**Figure 4:**
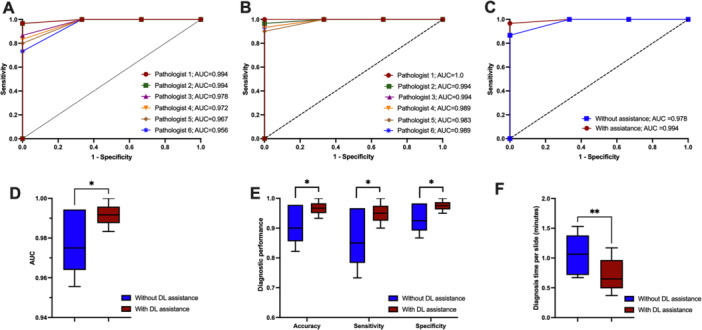
Performance of pathologists with or without DCNN assistance. (A) Receiver operating characteristic curves of six pathologists working alone, (B) receiver operating characteristic curves of six pathologists with DCNN assistance, (C) average receiver operating characteristic curves (ROC), (D) average area under curves (AUC), (E) accuracy, sensitivity, and specificity, and (F) average diagnosis time per slide. *p value < 0.05; **p value < 0.01.

To determine the potential use of DCNN in assisting pathologists when making diagnoses of BFOLs of the jaws, the performance of pathologists with or without DCNN assistance was further assessed. With the DL assistance, five out of six pathologists increased their accuracy in diagnosing BFOLs. DCNN significantly improved the pathologists’ diagnostic performance, with the average AUCs of pathologists with and without assistance being 0.994 and 0.978, respectively (*p* = 0.025). In addition, significantly greater accuracy (*p* = 0.025), sensitivity (*p* = 0.025), and specificity (*p* = 0.025) were observed when pathologists diagnosed with predicting results of DCNN. The DCNN assistance also helped increase the pathologists’ efficiency in diagnosing these lesions. On this data set of 30 microscopic slides, pathologists required an average of 32.0 ± 10.5 min to complete the diagnoses on their own, whereas, with the aid of DCNN, the diagnosis time was reduced to 21.3 ± 8.5 min (*p* = 0.007) (Figure 4).

## 4 Discussion

The present study is the first to test the DCNN for classifying BFOLs of the jaws and its application as an assisting tool to pathologists when making diagnoses of these lesions. We assessed five pre‐trained DCNN models, following various image pre‐processing and augmentation modes. These analyses highlighted the distinct capabilities and limitations of each DCNN model. The Xception model showed the highest overall accuracy based on the validation data set. However, the InceptionV3 model yielded better precision, recall, and F‐1 score. Other models, including the InceptionResNetV2, DenseNet201, and ResNetRS101, offered less robust performance in this setting and may be more suitable in specialized tasks where avoiding specific diagnostic errors is more crucial. Overall, the InceptionV3 model achieved the most balanced performance and was selected as the best model for BFOL classification. The superior performance of the InceptionV3 model in this study is likely due to its sophisticated multi‐scale feature extraction architecture, which simultaneously processes image information at different receptive field sizes. This capability is particularly valuable in histopathology, where diagnostic features range from detailed cellular morphology to broad architectural patterns, both of which are crucial for accurate BFOL classification.

The Inception and ResNet model families have established themselves as dominant architectures in histopathologic AI applications, with successful implementations across various diagnostic tasks, including cancer detection (Wang et al. 2023), tissue classification (Kumar et al. 2022), and morphometric analysis (Sharma et al. 2023). Their popularity stems from their ability to capture hierarchical feature representations while maintaining computational tractability. Based on our results and the broader literature, InceptionV3 represents an excellent choice for further histopathologic AI development, offering a well‐balanced combination of feature extraction capability, computational efficiency, and clinical applicability. However, the rapidly evolving landscape of DL architectures necessitates continued exploration of emerging models. Vision transformers and hybrid CNN‐Transformer architectures are increasingly demonstrating superior performance in medical imaging tasks, suggesting that future histopathologic AI systems may benefit from incorporating attention‐based mechanisms alongside or in place of traditional convolutional approaches. Therefore, while InceptionV3 serves as a reliable foundation, researchers should consider evaluating these recent architectures as computational resources and implementation frameworks continue to mature.

We also observed that the InceptionV3 model exhibited superior diagnostic accuracy in the test data set compared to the validation data set when assessing these lesions. This disparity may be attributed to the fact that the performance metrics derived from the validation data set in this study were computed from the individual image patches obtained for each case. Conversely, the test data set involved calculating the predicted diagnosis from the entire case by aggregating the predicted diagnoses of all image patches. This approach enabled the comparison of the DCNN performance with that of pathologists. These findings suggest that the accuracy of DCNN could be enhanced by providing more images of the cases and pooling the data for a comprehensive analysis of the entire case or slide.

When evaluating the performance of the DCNN and pathologists, we noted that the InceptionV3 model exhibited excellent performance in recognizing COF with 96.7% accuracy and a 95.2% F‐1 score, which was better than the average performance of pathologists (90.0% accuracy and 84.4% F‐1 score). However, the model performed inferiorly in distinguishing FD, compared with pathologists, with a relatively high number of false negative cases. In COD cases, the DCNN exhibited greater sensitivity but lesser specificity, constituting a slightly lower accuracy than pathologists. Our data suggest that the model tends to overcall COD but is too conservative in FD cases. This could be due to overfitting of the training data, leading to the diminished ability of the model to generalize features across lesions. This issue is one of the main challenges in developing DL algorithms that involve rare pathologic conditions (Litjens et al. 2017).

In contrast to the DCNN performance, pathologists demonstrated the highest diagnostic accuracy for FD, followed by COF and COD, respectively. This may result from the markedly overlapping histopathologic features between COF and COD (Su et al. 1997). Microscopically, COF and COD may show a mixture of different types of mineralized tissues (woven bone, lamellar bone, and cementum‐like material) in a variably cellular connective tissue stroma. In contrast, FD mainly comprises monotonous curvilinear woven bone trabeculae, making its microscopic distinction relatively more straightforward. In addition, these differences may partly be due to the setting of our study, where pathologists were able to examine the entire BFOL sections freely at different microscopic magnifications, whereas all patches of photomicrographs used to test the DCNN were obtained from the same magnification.

To explore the potential use of DCNN and its impact on pathologists' decision‐making, we compared the diagnostic performance of pathologists with and without DCNN assistance. Interestingly, we observed that with DCNN assistance, pathologists' average accuracy, sensitivity, and specificity in diagnosing BFOLs were significantly improved, with five out of six pathologists demonstrating increased overall diagnostic performance. When considering the different diagnoses between the two trials, we found that with DCNN assistance, pathologists changed their previous diagnoses to the correct ones 94.7% of the time. Notably, the cases where DCNN results most effectively enhanced the diagnostic accuracy of pathologists were between COF and COD, representing 73.7% of all differing diagnoses between the trials with and without DCNN assistance. This finding corresponds to the DCNN performance, which shows strong performance in distinguishing COF and COD, while pathologists are better at recognizing FD. These findings indicate that the DCNN influences pathologists to reassess uncertain cases, particularly between COF and COF, and helps make accurate diagnoses. In contrast, in circumstances where pathologists are confident in their diagnoses, such as in FD cases, the DCNN results do not alter pathologists' decisions. Therefore, our data suggest the DCNN could be helpful as an adjunctive tool since both DCNN and pathologists may succeed or fail in different situations. The cooperation between pathologists and the DCNN helps increase the diagnostic performance, compared with either pathologists or the DCNN working alone. In addition, we observed that the DCNN significantly reduced the time pathologists used to diagnose these lesions, indicating that AI could help improve efficiency when incorporated into the diagnostic pathology workflow.

Our study carries some limitations. The data set used in the present study was collected from a single institution. In pathology, multiple factors can affect the predicted outcomes of DL algorithms. Variations in color and quality of H&E‐stained slides may vary among laboratories, depending on the tissue fixation, specimen handling, processing, and staining techniques. In addition, we only include the three most commonly encountered BFOLs in the analyses. There are other bone pathoses, such as osteoma, osteoblastoma, juvenile trabecular ossifying fibroma, and psammomatoid ossifying fibroma, that show relatively similar histopathologic features and could be considered in the microscopic differential diagnoses in the clinical setting. Because of the rarity of these lesions and the inherent data set‐related variations, future collaborative studies to assemble extensive data on fibro‐osseous and other related lesions from multicentric institutions should be beneficial. The expanded lesion types and sample size could help increase the data set diversity and improve the generalizability of the DL algorithms. Furthermore, our study only examines the histopathologic aspects of BFOL diagnosis, whereas clinical and radiographic information is also crucial in practice. Therefore, developing and incorporating the radiographic data in the DCNN may also help refine the diagnostic accuracy of these lesions.

## 5 Conclusions

The DCNN can potentially be used to distinguish the three most common BFOLs of the jaws. Incorporating the developed DL assistance effectively improves pathologists' diagnostic accuracy while reducing time. Our results may serve as a basis for the evolving development of AI that could help revolutionize the future practice of diagnostic pathology.

## Author Contributions

Conceptualization and study design: all authors. Data curation, investigation, and analysis: Paniti Achararit and Ekarat Phattarataratip. Project administration and supervision: Ekarat Phattarataratip, Paniti Achararit, Kraisorn Sappayatosok, Thanaphum Osathanon, Chawan Manaspon, and Chavin Jongwannasiri. Writing – original draft: Paniti Achararit and Ekarat Phattarataratip. Writing – review and editing: Paniti Achararit, Ekarat Phattarataratip, Kraisorn Sappayatosok, and Thanaphum Osathanon. Approval of final manuscript: all authors.

## Ethics Statement

This study was approved by the Faculty of Dentistry, Chulalongkorn University Human Research Ethics Committee (DCU 2022‐089) and performed according to the Declaration of Helsinki. The requirement for informed consent was waived by the Institutional Review Board.

## Consent

The authors have nothing to report.

## Conflicts of Interest

The authors declare no conflicts of interest.

## Data Availability

The data that support the findings of this study are available from the corresponding author upon reasonable request.
